# Marketing to the ‘Right’ Older Adult: Whose Aging is Represented in Recruitment Materials?

**DOI:** 10.1093/geroni/igaf122.3975

**Published:** 2025-12-31

**Authors:** Regan Metz, Kyra Corbisiero, Abigail LeGars, Kaitlyn Langendoerfer

**Affiliations:** Wilkes University, Wilkes-Barre, Pennsylvania, United States; College of the Holy Cross, Worcester, Massachusetts, United States; Wilkes University, Wilkes-Barre, Pennsylvania, United States; Wilkes University, Wilkes-Barre, Pennsylvania, United States

## Abstract

There are nearly 11,000 older adult centers in the US. While well attended by previous generations, as more baby boomers aged, most centers experienced a declining clientele. These centers have begun to reconceptualize their role to service the interests of the new cohort. They now include cafes, wellness programing, and lifelong learning. This begs the question, who are older adult centers recruiting and what are the potential impacts? This study was designed to examine older adult center promotional materials using content analysis of the images featured within brochures, newsletters, and social media pages from 140 centers located in Pennsylvania. Three raters independently coded the images on a range of variables including observed demographics, health status, physical limitation, appearance (e.g. wrinkles/gray hair), and overall portrayal of aging. Our findings demonstrated that promotional materials reflected the demographics of Pennsylvania’s aging population in terms of race and gender. However, the images overwhelmingly portrayed older adults as healthy (93%), happy (71%), and without any significant signs of decline; emphasizing a general image of “successful aging.” Notably, only 40% of images were coded as representing “inclusive aging,” meaning that most promotional materials do not accurately reflect the broader realities and diversity of the overall aging population. Failing to promote inclusivity, marketing materials currently frame the centers as recreation-style clubs, rather than spaces supportive of underrepresented groups. Discussed are the impacts on public funding of older adult centers and on marginalized community-dwelling older adults more generally.

